# Monotherapy to Polytherapy: Antiepileptic Drug Conversions Through the Spectrum of Epilepsy Care

**DOI:** 10.2174/157015909788848910

**Published:** 2009-06

**Authors:** Erik K St. Louis

**Affiliations:** Mayo Clinic, Rochester, Minnesota, USA

**Keywords:** Epilepsy, antiepileptic drugs, conversion, monotherapy, polytherapy.

## Abstract

The process of conversion between AED monotherapies is frequently necessary in epilepsy care, yet little practical guidance is available to practitioners. This article introduces an issue of *Current Neuropharmacology *devoted to the theme of AED conversions and related issues. In this series of articles, we reviewed the role of AED monotherapy in newly diagnosed epilepsy, the practice of transitional polytherapy during AED monotherapy conversions in patients experiencing breakthrough seizures or adverse effects, chronic maintenance polytherapy for refractory epilepsy, and the related topics of strategies for minimizing adverse effects, appropriate blood level monitoring, and patient-related factors in AED conversions. Successful conversion between AED monotherapies and polytherapy drug sequencing requires that practitioners possess and apply a thorough knowledge of epilepsy, AED pharmacology, and clinical reasoning, while being sensitive and reactive to patient reported adverse effects of treatment.

“One thing at a time, all things in succession. That which grows fast, withers as rapidly. That which grows slowly, endures.”

Dr. Josiah Gilbert Holland, Founder of Scribner’s Monthly

“When the music changes, so does the dance.”

African Proverb

## INTRODUCTION

The first antiepileptic drug (AED) monotherapy utilized successfully manages nearly half of epilepsy patients; however, conversion to a second monotherapy is necessary for those who fail to become seizure-free or who do not tolerate an initially chosen AED, and chronic polytherapy is necessary in many patients who develop refractory epilepsy [1]. Over the last two decades, there has been more progress in available treatments for epilepsy than any other time in history. There are now 17 marketed commonly used AEDs, eleven of which were approved since 1990. While all newer AEDs were first approved in the United States for adjunctive treatment of partial-onset seizures—leading to their near-exclusive use as polytherapy initially—there is now increasing use of newer AEDs as monotherapy paralleling expanding evidence basis for formal approval of monotherapy use, and adequate evidence to support off-label monotherapy use. Increasing generic availability may further encourage earlier use of newer AEDs in both monotherapy and adjunctive therapy situations. Given this shifting paradigm in uses of newer AEDs from sole polytherapy applications to increasing monotherapy usage, and the practical need to transition AEDs in many patients, the subject matter of conversions between AED monotherapies is timely.

Monotherapy is widely favored over polytherapy by neurologists in the treatment of newly diagnosed epilepsy, limited evidence for this “monotherapy maxim” notwithstanding. Abundant evidence is available when initiating adjunctive AED therapy in refractory epilepsy, on practical grounds an important setting for use of newer AEDs since when a new AED becomes available, it is generally first limited to use in refractory patients having the greatest need for improved seizure control, until its safety and tolerability profile and expanded evidence for use in other settings is well established. While it is probable that all AEDs proven effective in adjunctive polytherapy settings are also efficacious for use as monotherapy, available evidence guiding AED monotherapy remains somewhat limited, leading to relatively narrow FDA indications in United States practice. Several newer AEDs have randomized controlled trial evidence for monotherapy application, while clinical experience and lesser evidence lead to FDA “grandfathering” of older AEDs for monotherapy indication in newly diagnosed epilepsy. However, relatively little evidence is available to guide clinicians as they implement changes between monotherapy or polytherapy AED regimens for patients with epilepsy. A panel of epilepsy neurologists and clinical pharmacologists was recently convened to address this important topic. The goal of the SPECTRA panel (Study by a Panel of Experts: Considerations for Therapy Replacement in Antiepileptics) was to develop consensus regarding practical issues in AED monotherapy conversions. This panel developed consensus on a key overriding treatment principle: to fully titrate an adjunctive drug prior to tapering a baseline drug when possible. The panel also developed drug-specific consensus recommendations that expand and complement available prescribing information that is useful in planning AED monotherapy conversions as well as AED sequencing in chronic polytherapy regimens [2].

This issue of *Current Neuropharmacology* focuses on the topic of AED conversions in epilepsy care. This series of articles begins with a practical review of AED monotherapy in newly diagnosed epilepsy, next examines factors involved in monotherapy conversions during the process of transitional polytherapy, then outlines important considerations in ensuring successful chronic polytherapy. A discussion of the rationale and strategies for minimizing adverse effects in epilepsy care is then followed by a review of principles for appropriate blood level monitoring. Finally, this issue considers important patient-related factors in AED conversions. The above-referenced motivational sentiment of 19^th^ century physician, author, and poet Josiah Holland well anticipated the approach to epilepsy treatment favored herein: initiate AED treatment as monotherapy, sequence medications as needed to achieve seizure control or improve a patient’s acceptance and tolerability of therapy, and titrate most medications slowly and carefully to ensure maximal success. However, as the ensuing proverb reminds us, conversion between different AEDs may require specific modifications to ensure treatment success. The SPECTRA data summarized herein provides clinicians with a helpful new approach to guide conversions between AED therapies, and the accompanying review articles offer further relevant guidance for clinicians implementing AED conversions in epilepsy care.
